# Mineral and Microbiological Analysis of Spices and Aromatic Herbs

**DOI:** 10.3390/foods11040548

**Published:** 2022-02-15

**Authors:** Nicola Cicero, Teresa Gervasi, Alessandra Durazzo, Massimo Lucarini, Antonio Macrì, Vincenzo Nava, Filippo Giarratana, Roberta Tardugno, Rossella Vadalà, Antonello Santini

**Affiliations:** 1 Department of Biomedical and Dental Sciences and Morphofunctional Imaging, University of Messina, 98168 Messina, Italy; ncicero@unime.it (N.C.); macri.anto@outlook.it (A.M.); vnava@unime.it (V.N.); rosvadala@tiscali.it (R.V.); 2 Science4life, Spinoff Company, University of Messina, 98168 Messina, Italy; roberta.tardugno@gmail.com; 3 CREA—Research Centre for Food and Nutrition, Via Ardeatina 546, 00178 Rome, Italy; alessandra.durazzo@crea.gov.it (A.D.); massimo.lucarini@crea.gov.it (M.L.); 4 Department of Veterinary Sciences, University of Messina, 98168 Messina, Italy; fgiarratana@unime.it; 5 Department of Pharmacy, University of Napoli Federico II, Via D. Montesano 49, 80131 Napoli, Italy

**Keywords:** spices, aromatic herbs, minerals, chemical analysis, microbiological analysis

## Abstract

Spices and aromatic herbs have always had great historical importance in human nutrition. Their use has been documented for centuries as a rich source of bioactive compounds; they have been used for their health benefits and also for flavoring or coloring food. However, despite the many health properties linked to the use of spices and aromatic herbs, these can represent biological hazards and can contain chemical substances of concern. Certainly, monitoring potential health hazards in spices and aromatic herbs includes microbiological safety and also the content of inorganic substances: both represent a key step. This research aims at monitoring the compliance of various spices and aromatic herbs from a non-European country market (namely: black cumin seeds, Iranian Tokhme Sharbati, clove buds, Shahjeera, Abbaszadeh saffron, organic fenugreek, whole black pepper, cinnamon, Abthul Ahmar (Asario), Ajwan seeds, whole coriander seeds, black sesame seeds, Sabja seeds) with the current European Union (EU) and WHO regulations, when available, regarding mineral and microbiological parameters. In particular, microbiological assays using rapid and conventional methods, and trace mineral determination by inductively coupled plasma mass spectrometry (ICP-MS) were performed. Results show the safety of the tested spices, given that the microbiological parameters were within the legal microbiological criteria set by the European Commission Regulation (EC) No. 2073/2005 and its amendment Regulation (EC) No. 1441/2007. With reference to potentially toxic Cd, Pb, As, Hg, these were within the limits set by the European Commission Regulation (EC) No. 1881/2006 and its amendments, Regulation (EU) No. 1317/2021 and Regulation (EU) No. 1323/2021, and WHO. According to EU regulations, for Pb content, 2 samples out of 16 showed values different from the set limits.

## 1. Introduction

Spices and aromatic herbs have always had great historical significance in human nutrition and in holistic approaches to health issues. In fact, they were used in ancient times not only in the food sector, i.e., to flavor and aromatize dishes, but also in the medical field, in many religious rituals, and for the preservation of food. They are documented as rich source of bioactive compounds linked to health benefits [1]. However, vegetals can be contaminated by microorganisms and can accumulate heavy metals, pesticide residues, and other potentially toxic substances from the environment depending on environmental factors, e.g., soil characteristics and absorbability, water, air, plant genotypes, and anthropogenic activities [2,3,4,5,6,7,8,9].

Acute or chronic poisonings may occur following heavy metal intake through food. Their bioaccumulation may lead to diverse toxic effects on a variety of body tissues and organs. Heavy metals disrupt cellular events including growth, proliferation, differentiation, damage-repairing processes, and apoptosis [10].

Thus, spices and aromatic herbs can be biological and chemical threats when used as food ingredients or for medicinal uses [11,12,13]. The conservation of spices and aromatic herbs is carried out in most cases via the dehydration process, through physical processes such as heat and/or pressure. This conservation methodology is minimally invasive and aims to inhibit metabolic activities, and therefore the proliferation of microorganisms by subtracting the free and bound water within the food. Dehydrated foods are characterized by a low moisture content (<14%), corresponding to a low value of free water with water activity (A_w_) that is less than 0.75. Compared to the native product, the reduction in A_w_ values therefore represent one of the main parameters of ensuring the inhibition of the growth of organisms, consequently giving stability to the food from both a microbiological, enzymatic, and chemical point of view. Foodstuffs of vegetable origin such as cereals and derivatives, seeds, dried fruit, coffee, cocoa, herbs, and spices are easily attacked by molds, which in particular conditions of temperature and humidity, can produce secondary potentially dangerous metabolites such as mycotoxins [14,15,16,17,18].

Although spices and aromatic herbs on the market are dehydrated products and therefore have stable chemical and physical characteristics, these foods are generally subjected to drying processes at room temperature in their places of origin, which generally are developing or tropical countries where the production technologies used are not always capable of guaranteeing the implementation of good hygiene and safety practices, thus becoming the main cause of contamination [19,20]. Nevertheless, even with low water activity levels, some microorganisms, including pathogenic and toxigenic ones, are able to survive and may proliferate when vegetal matrices are added to foods.

Considering the crucial role of safety and quality in food production, in Europe, systems to detect and neutralize contaminants in herbs and spices have been developed within the project “Securing the spices and herbs commodity chains in Europe against deliberate, accidental, or natural biological and chemical contamination” (SPICED). The EU market is one of the main world markets for spices and herbs, and the problem linked to microbiological and toxicological hazards can pose a serious risk for the consumer, as spices and herbs could potentially contaminate a wide range of products due to their widespread use. Europe is one of the most important regions in the world with reference to the importation of herbs and spices, accounting for about one quarter of the world’s total imports of herbs and spices [21,22].

Different microorganisms could be potentially harmful in herbs as well as in different food matrices, i.e., *Staphylococcus aureus*, *Salmonella* spp., *Escherichia coli*, *Listeria monocytogenes*, aflatoxin-producing fungi (i.e., *Aspergillus* spp.), *Clostridium perfringens,* and *Bacillus cereus* [16,23,24,25].

Taking into account these possible threats to health, the problem of spice and herb product consumption has global significance. 

There are many studies on microflora in agricultural products, but only a small part of them address spices and officinal and aromatic herbs, which are instead increasingly present in our daily diet, thus also acquiring an increasingly important economic role. 

On the other hand, monitoring the presence of metals in spices and herbs represents a key step [25,26,27].

Metals have important biological functions and activities, but inorganic elements can become toxic when their intake exceeds the accepted and maximum allowed levels as suggested by the European Commission and the European Food Safety Authority (EFSA) [28,29,30,31,32,33,34,35,36]. 

Considering that Europe is among the main importer of spices from extra EU countries [21,22], monitoring the contaminant levels in spices and herbs from these countries may provide relevant toxicological data on spices and aromatic herbs commonly present in the European Union (EU) market, improving the accuracy of dietary risk exposure/assessment, and thus enhancing the feasibility of epidemiological studies. In this context, the present study aims to monitor the safety of some spices and herbs from foreign countries by evaluating: (i) microbiological contamination, and (ii) trace mineral element content. 

## 2. Materials and Methods

### 2.1. Samples

Spices and aromatic herbs of different species, and of different countries of origin, were acquired in an international market in Saudi Arabia. The spices and herbs that were the object of this study are among the main ones imported into Europe and distributed in European countries. In this context, the end points of the present study were to check conformity with existing EU regulations, and the content of trace metal elements.

Thirteen samples of spices and aromatic herbs (labelled from A to O), packed in sealed plastic bags, were subjected to microbiological and chemical analysis. Table 1 reports the description of the samples according to their scientific name, vulgaris name, and country of origin. All microbial and chemical analyses have been carried out in triplicate.

### 2.2. Microbiological Methodology

The counts of total mesophilic bacteria, fungi (yeasts and molds), total coliforms, *Escherichia coli*, Enterobacteria, lactic bacteria, *Staphylococcus aureus*, *Salmonella* spp., *Listeria monocytogenes*, *Bacillus cereus*, and sulphite-reducing clostrides were performed. All media were supplied by ThermoFisher Scientific, Oxoid Ltd., Basingstoke, UK. The samples were homogenized with PBS (pH = 7.4) by mixing vigorously. Then serial dilutions in PBS (pH = 7.4) were performed, and the samples were spread aseptically over the media plates. For mesophilic counts, samples were spread over plate count agar (PCA) and incubated at 30 °C for 72 h. Malt extract agar with 10% lactic acid (MEA) was utilized to verify the counts of yeasts and molds by incubating at room temperature for 3 days. Lactic acid microorganisms and Enterobacteriacee were investigated using De Man, Rogosa, and Sharpe agar and Violet Red Bile Glucose Agar (MRSA and VRBG) using an incubation period of 72 h at 30 °C and 48 h at 37 °C, respectively. The determination of *B. cereus* was carried out by using *Bacillus Cereus* Agar Base (PEMBA) after an incubation for 24 h at 37 °C. Coliforms and *E. coli* were analyzed using the standard membrane filter technique using Chromogenic Coliform Agar (CCA) and Tryptone Bile X-Gluc agar (TBX) using an incubation period of 24 h at 37 °C and of 18/24 h at 44 °C. *S. aureus* contamination was detected using the standard membrane filter technique using Baird–Parker Agar (BPA), at 37 °C for 48 h. The standard membrane filter technique using Sulfite Polymyxin Sulfadizine agar (SPS) was used for the determination of sulphite-reducing clostrides after an incubation at 37 °C for 48 h. The determination of *L. monocytogenes* was carried out according to ISO standard 11290. Briefly, after a two-stage enrichment process, the first in half Fraser broth for 24 h and then in Fraser broth, the enriched broths were plated on Oxford and BD PALCAM *Lysteria* agars.

The presence of *Salmonella* spp. was investigated according to ISO standard 6579, which includes: pre-enrichment in a non-selective liquid medium (buffered pepton water), followed by selective enrichment (Rappaport–Vessiliadis Soy Broth; ThermoFisher Scientific, Oxoid Ltd., Basingstoke, UK), and then isolation on a selective medium (Hektoen; ThermoFisher Scientific, Oxoid Ltd., Basingstoke, UK). All microbial analyses were carried out in triplicate. 

### 2.3. Mineral Analysis

#### 2.3.1. Sample Preparation

A total of 0.25 g dry weight (dw) of each sample was milled in a Teflon mortar and digested with 7 mL of 65% (*v*/*v*) HNO_3_, and 1 mL of 30% (*v*/*v*) H_2_O_2_ (J.T. Baker, Mallinckrodt Baker, Milan, Italy). A total of 1 mL of 0.8 mg L^−1^ Re (Fluka, Milan, Italy) was added as an internal standard. Mineralization was performed in an Ethos 1 digestor (Milestone, Bergamo, Italy) at 1000 W and 180 °C; this temperature was reached in 10 min and held for another 10 min. The digested samples, cooled at room temperature, were diluted with ultrapure deionized water obtained (J.T. Baker, Mallinckrodt Baker, Milan, Italy) and stored at 4 °C. 

#### 2.3.2. ICP-MS Analysis

Minerals were determined by the same procedure utilized for the determination of potentially toxic inorganic species in vegetables as reported in a previous work [16]. An Agilent 7500CX ICP-MS spectrometer (Agilent, Santa Clara, CA, USA) equipped with an Octapole Reaction System (ORS) reaction/collision cell, and with an ASX 500 auto sampler, was used for analyzing the digested samples. The system was pressurized with 99.9% pure helium (Rivoira S.p.A., Milan, Italy). 

The operating conditions of the ICP-MS were as follows: RF power, 1550 W; plasma gas flow rate, 15 L min^−1^; auxiliary gas flow rate, 0.9 L min^−1^; carrier gas flow rate, 1.1 L min^−1^; sample introduction flow rate, 1 mL min^−1^; sample depth, 9 mm; spray chamber temperature 2 °C; vacuum, <1.5 × 10^−7^ Pa; interface pressure, 5.3 × 10^−2^ Pa.

^7^Li, ^59^Co, ^80^Y, and ^205^Tl solutions (Agilent, Santa Clara, CA, USA) at a concentration of 10 μg L^−1^ were used for tuning the instrument. 

^63^Cu, ^60^Ni, ^75^As, ^51^V, ^52^Cr, and ^208,207,206^Pb (Fluka, Milan, Italy), and ^114^Cd and ^202^Hg isotopes (Merck, Darmstadt, Germany) were selected to optimize the sensitivity, and to minimize matrix interference. 

A solution of ^115^In, ^45^Sc, ^103^Rh, and ^209^Bi at a concentration of 10 µg L^−1^ was used as an online internal standard to correct any instrumental drifts and matrix effects.

Quantitative determinations were performed using the external standard method. The calibration used a multi-standard solution of Cr, V, Cu, Cd, Pb, and Ni at different concentrations ranging from 0.5 to 2000 μg L^−1^.

Hg was analyzed separately following a previously described procedure [5]. All analyses were carried out in triplicate.

#### 2.3.3. Statistical Analysis

All mineral data are reported as the mean and standard deviation of three independent determinations. With regards to the selected heavy metals (Cd, Pb, As, and Hg), their contents were statistically compared with the reference limit values established by the European Commission and WHO by means of Student’s t test. The statistical analysis has been performed using the Past software (Version 4.09) [37].

## 3. Results and Discussion

The safety aspects that were explored throughout the study were: (i) microbiological aspects; and (ii) mineral profiles.

### 3.1. Microbiological Analysis

The microbiological analysis of 13 dried spices, labelled with letters from A to O, is reported in Table 2. 

According to the Commission Regulation (EC) No. 2073/2005 and its amendment Regulation (EC) No. 1441/2007, which sets legal microbiological criteria for several food products, these spices could be defined safe products [8,38,39].

With regards to the indicator microorganisms, which are used to provide simple, reliable, and rapid information about processing failures, post-processing contamination from the environment, the general level of hygiene, and the presence or absence of foodborne pathogens to monitor the chain of food production, low bacterial loads were detected in saffron, fenugreek, black pepper, cinnamon, cress sprouting seed, thymol seed, coriander, and chia seed [40].

The total coliform and Enterobacteriaceae amounts in black cumin were 7 × 10^3^ CFU g^−1^ and 1.4 × 10^4^ CFU g^−1^, respectively, whereas in Iranian chia seeds it was 1.6 × 10^3^ CFU g^−1^ and 6.6 × 10^2^ CFU g^−1^, respectively. Caraway presented a mesophilic count of 1.7 × 10^6^ CFU g^−1^, and an amount of total coliform and Enterobacteriaceae of 2.4 × 10^4^ CFU g^−1^ and 9 × 10^4^ CFU g^−1^, respectively. Moreover, Enterobacteriaceae were also detected in clove buds (2 × 10 CFU g^−1^) and black sesame seeds (4.4 × 10^3^ CFU g^−1^). 

These contaminations may be related to the environment, inadequate hygienic handling, unsanitary conditions, and others, and occurs in samples purchased in street markets. Indeed, the adoption of good hygiene practices in all the involved steps from land growing, harvesting, and processing can be useful to reduce the health risks of spice consumption.

### 3.2. Mineral Analysis

#### 3.2.1. Method Validation

The method was validated according to Eurachem criteria [41]. Commercial standards were used for the evaluation of method linearity, limits of detection (LODs), limits of quantification (LOQs), accuracy, repeatability, and intermediate precision. LODs and LOQs were calculated as 3.3 σ/S and 10 σ/S, respectively, where σ is the standard deviation of six blanks and S is the slope of the relative calibration curve. A good linearity was obtained for all elements investigated with R2 values ranging from 0.9992 (for Cu and Se) to 0.9999 (for V). The limits of detection (LODs) ranged from 0.001 to 0.051 µg kg^−1^, and the limits of quantification (LOQs) ranged from 0.003 to 0.168 µg kg^−1^. The lowest average recovery was observed for mercury with 92.93%, while the highest was obtained for strontium with 103.03%. Accuracy was assessed by evaluating six determinations on certified reference materials (NIST1570A spinach leaves) and was reported as the percent recovery between the value found with the calibration curve and the true value reported in the certified reference materials. If the element was not certified in the reference materials, the matrix was spiked with the known amount of analyte, and was analyzed following the procedures discussed previously. Based on these results, the analytical characteristic (linearity, sensitivity, and accuracy) can be considered to be satisfactory for the purposes of the analysis (Table 3). 

#### 3.2.2. Mineral Contents

The content of inorganic elements in the samples, labelled with letters from A to O, are shown in Table 4 and reported as average values and standard deviations. 

Although various classifications for trace elements have been proposed and may be controversial, this paper uses the World Health Organization recommendations which classifies trace elements as: essential trace elements, potentially non-toxic essential elements, and potentially toxic elements [42].

#### 3.2.3. Trace Elements

Given the increasing consumption of spices and aromatic herbs in the daily diet, it is also interesting to elucidate the content of minerals known for their nutritional roles such as Cr, Fe, Mn, Cu, Se, and Zn. Several studies on the content of mineral and trace elements in spices and herbs have remarked that they occur in a wide range of concentrations [43,44,45,46]. 

Data on Cr content in these products are needed. Chromium has been quantified in several foods and beverages, and currently the most comprehensive source are the Danish food composition tables [47]. The content of chromium in foods is relatively low and most foods present a content below 0.1 mg kg^−1^. Data from the literature indicate that the presence of Cr in spices and aromatic herbs is higher than other foods and beverages, within a range from 0.01 to 3.0 mg kg^−1^ [48,49,50,51]. In our study, the chromium level found in spices was below 0.3 mg kg^−1^, except for the high content found in cinnamon (2.35 mg kg^−1^) and thymol seeds (1.11 mg kg^−1^). 

Selenium content varied from 0.003 to 0.505 mg kg^−1^; in particular, selenium was reported with values: 0.254, 0.287, 0.254, 0.505, and 0.485 mg kg^−1^ for caraways, cress sprouting seeds, thymol seeds, coriander, and black sesame seeds, respectively. Selenium (Se) is an essential trace element involved in the synthesis of various selenium-containing proteins, and also has other relevant biological functions; moreover, it has a fundamental role in the human diet since it may act as a preventive agent against some health conditions [51,52]. 

#### 3.2.4. Potentially Non-Toxic Elements

Eight trace elements that are not normally known for their toxic effects were identified, but it is nonetheless important to monitor them, because if very high concentrations of these metals are ingested, they can lead to physiological disorders [53,54]. Their maximum concentrations were found to be in the decreasing order as follows: Sr > Ni > Sn > V > Co > Sb > Be. All these potentially non-toxic trace elements were contained in variable amounts in the analyzed spices. In particular among these, the major trace elements were: Sr, which ranged from 95.92 ± 0.20 mg kg^−1^ (carraway) to 4.30 ± 0.02 mg kg^−1^ (fenugreek); Ni, which ranged from 3.05 ± 0.12 mg kg^−1^ (black cumin) to 0.42 ± 0.01 mg kg^−1^ (chia seeds); Sn, which ranged from 2.39 ± 0.04 mg kg^−1^ (coriander) to 0.11 ± 0.04 mg kg^−1^ (clove buds); and Co, which ranged from 0.29 ± 0.00 mg kg^−1^ (thymol seeds) to 0.02 ± 0.00 mg kg^−1^ (black pepper).

#### 3.2.5. Potentially Toxic Elements

Concerning Cd, Pb, As, and Hg, the following ranges have been observed in the 13 analyzed spices: Cd (0.003–0.079 mg kg^−1^), Pb (0.008–0.544 mg kg^−1^), As (0.003–0.339 mg kg^−1^), and Hg (0.001–0.010 mg kg^−1^). The contamination level of the analyzed samples followed the sequence: Pb > As > Cd > Hg.

Heavy metals should be closely monitored, considering that the absorption and bioaccumulation of those compounds, with reference to their toxic and mutagenic effects, have a negative effect on consumers’ health [8,44,45,55]. 

According to the European Commission Regulation (EC) No. 1881/2006 and its amendments, Regulation (EU) No. 1317/2021 and Regulation (EU) No. 1323/2021, the maximum levels in fresh herbs for lead and cadmium have been set at 0.1 mg kg^−1^ and 0.2 mg kg^−1^, respectively. However, for many elements, there is a lack of shared worldwide regulation, and reference could be made to the values reported by WHO and EFSA, namely 5.0 and 0.2 mg kg^−1^ for As and Hg, respectively [28,29,30,31,32,33,34,35,36,56,57].

Concerning the content of potentially toxic minerals (Cd, Pb, As, and Hg), the results showed that all the spices analyzed did not present contamination concerns for cadmium, arsenic, and mercury, as the contents of these three metals were always lower than the permitted limit values established by the European Commission regulation and WHO. On the other hand, for Pb content, two samples showed two warning values with respect to the established permitted limit of 0.1 mg kg^−1^. Ajwan seeds from India (sample L) showed a lead content (0.544 mg kg^−1^) five times higher than the permitted limit (*p* < 0.05) while the lead content found in Abbaszadeh saffron from Iran (0.096 mg kg^−1^, sample E) was close to the accepted limit (*p* > 0.05), indicating that for these samples, there was a possible threat to health.

Several studies have reported a potential threat to the nervous system from aluminum [58,59]. The content of aluminum ranges from 7.112 mg kg^−1^ in fenugreek to 46.889 mg kg^−1^ in chia seeds, with the exception of carraway, saffron, and thymol seeds, in which the reported values were 87.507 mg kg^−1^, 145.216 mg kg^−1^, and 930.198 mg kg^−1^, respectively, showing a high capacity to accumulate aluminum.

Lopez et al. [58] showed data on the levels of Al in 72 dried samples of 17 different spices and aromatic herbs, and aluminum levels ranged from 3.74 to 56.50 mg kg^−1^. Bratakos et al. found Al values in spices with a mean value of 157 mg kg^−1^ [58]. For foods from plant origins, high aluminum concentration could be related to its high content in the soil where the plants are grown, or to the fact that plants grow in acid soils, because its availability depends on soil pH [59,60]. 

## 4. Conclusions

This study aims at monitoring the levels of both microbial contamination and trace elements in some spices and aromatic herbs commonly used in the Mediterranean diet. The data indicate that black cumin and Iranian chia seeds presented contamination (total coliform bacteria and Enterobacteriaceae). The concentration of trace elements was variable and related to each spice. Concerning the contents of potentially toxic heavy metals (Cd, Pb, As, and Hg), they were within the above-mentioned limits, although Pb presented a higher value in two cases. 

It should be considered as a final remark that all spices and herbs are susceptible to environmental (e.g., microbial and heavy metal) contamination. Microbial contamination could be prevented by adopting good standards of practice during growing, harvesting, and processing. Environmental contamination with heavy metals should be avoided and monitored to minimize contamination levels. For this reason, spices and aromatic herbs must be strictly monitored for the aspects concerning their safety in order to prevent foodborne illness due to contamination.

## Figures and Tables

**Table 1:** Analyzed herbs and spices and their origin.

Label	Origin	Common Name	Scientific Name
Black Cumin Seeds (A)	India	Black cumin	*Nigella sativa*
Iranian Tokhme Sharbati (B)	Iran	Chia seeds	*Salvia hispanica*
Clove Buds (C)	Indonesia	Clove buds	*Syzygium aromaticum*
Shahjeera (D)	India	Caraway	*Carum carvi*
Abbaszadeh Saffron (E)	Iran	Saffron	*Crocus sativus*
Organic Fenugreek (F)	India	Fenugreek	*Trigonella foenum-graecum*
Whole Black Pepper (G)	Vietnam	Black pepper	*Piper nigrum*
Cinnamon (H)	Indonesia	Cinnamon	*Cinnamomum verum*
Abthul Ahmar (Asario) (I)	India	Cress Sprouting Seeds	*Lapidium sativum*
Ajwan Seeds (L)	India	Thymol seeds	*Trachyspermum ammi*
Whole Coriander Seeds (M)	India	Coriander	*Coriandrum sativum*
Black Sesame Seeds (N)	India	Black sesame seeds	*Sesamum indicum*
Sabja Seeds (O)	India	Chia seeds	*Salvia hispanica*

**Table 2:** Bacterial contamination (CFU g^−1^) of dried spices from India (**a**) and Iran, Indonesia, and Vietnam (**b**).

(a)									
Microorganisms	A	D	F	I	L	M	N	O	Reference Values ^a^
Mesophilic Bacteria	2.1 × 10^4^	1.7 × 10^6^ *	3.7 × 10^2^	30	1.6 × 10^3^	2.4 × 10^2^	3.2 × 10^2^	8.5 × 10^4^	<5 × 10^5^
Molds	1.2 × 10^2^	2.3 × 10^2^	1∙10	ND	ND	ND	1.2 × 10^2^	ND	<1 × 10^3^
Yeasts	30	7.1 × 10^2^	ND	ND	ND	ND	ND	ND	<1 × 10^3^
Coliforms	7 × 10^3^ *	2.4 × 10^4^ *	ND	ND	ND	ND	6.2 × 10^3^	ND	<1 × 10^3^
*Escherichia coli*	ND	ND	ND	ND	ND	ND	ND	ND	<10
Sulfite-Reducing Clostridia	ND	ND	ND	ND	ND	ND	ND	ND	<1 × 10^2^
Clostridial Spores	ND	ND	ND	ND	ND	ND	ND	ND	<1 × 10^2^
*Staphilococcus aureus*	ND	ND	ND	ND	ND	ND	ND	ND	<1 × 10^2^
*Salmonella* spp. ^b^	ND	ND	ND	ND	ND	ND	ND	ND	0
*Listeria monocytogenes*	ND	ND	ND	ND	ND	ND	ND	ND	<10^2^
*Bacillus cereus*	ND	ND	ND	ND	ND	ND	2 × 10	ND	<1 × 10^3^
*Enterobatteriacee*	1.4 × 10^4^ *	9 × 10^4^ *	ND	ND	ND	ND	4.4 × 10^3^ *	ND	<10
Lactic Bacteria	ND	ND	ND	ND	ND	ND	ND	ND	<1 × 10^5^
**(b)**									
**Microorganisms**		**B**	**C**	**E**	**G**	**H**	**I**	**Reference Values ^a^**	
Mesofilic Bacteria		6 × 10^4^	2.2 × 10^2^	2.5 × 10^2^	9.3 × 10^4^	1.1 × 10^2^	30	<5 × 10^5^	
Molds		2.5 × 10^2^	ND	ND	ND	ND	ND	<1 × 10^3^	
Yeasts		ND	ND	ND	ND	ND	ND	<1 × 10^3^	
Coliforms		1.6 × 10^3^ *	1.7 × 10^2^	ND	4∙10	ND	ND	<1 × 10^3^	
*Escherichia coli*		ND	ND	ND	ND	ND	ND	<1 × 10	
Sulfite-Reducing Clostridia		ND	ND	ND	ND	ND	ND	<1 × 10^2^	
Clostridial Spores		ND	ND	ND	ND	ND	ND	<1 × 10^2^	
*Staphilococcus aureus*		ND	ND	ND	ND	ND	ND	<1 × 10^2^	
*Salmonella* spp. ^b^		ND	ND	ND	ND	ND	ND	0	
*Listeria monocytogenes*		ND	ND	ND	ND	ND	ND	1 × 10^2^	
*Bacillus cereus*		ND	ND	ND	ND	ND	ND	<1 × 10^3^	
*Enterobatteriacee*		6.6 × 10^2^ *	2 × 10 *	ND	ND	ND	ND	<10	
*Lactic Bacteria*		ND	ND	ND	ND	ND	ND	<1 × 10^5^	

^a^ Reference values for microbiological safety and quality were based on regulations from the European Union and other international guidelines [8,38,39]. ^b^ CFU 25 g^−1^. * Values are higher than the reference values. ND, not determined. A, black cumin seeds; B, Iranian Tokhme Sharbati; C, clove buds; D, Shahjeera; E, Abbaszadeh saffron; F, organic fenugreek; G, whole black pepper; H, cinnamon; I, Abthul Ahmar (Asario); L, Ajwan seeds; M, whole coriander seeds; N, black sesame seeds; O, Sabja seeds.

**Table 3:** Analytical parameters for method validation.

Element	LOD (µg kg^−1^)	LOQ (µg kg^−1^)	R^2^	Experimental Value (mg kg^−1^)	Expected Value (mg kg^−1^)	Recovery (%)
Be	0.001	0.003	0.9998	1.90 *	2.00 *	95.00
Sn	0.005	0.017	0.9997	1.97 *	2.00 *	98.50
Al	0.051	0.168	0.9995	308.55	310.00	99.53
V	0.003	0.010	0.9999	0.575	0.568	101.23
Cr	0.003	0.010	0.9997	1.98	2.00 *	99.00
Mn	0.015	0.050	0.9996	75.3	76.0	99.08
Fe	0.035	0.116	0.9994	2.02	2.00 *	101.00
Co	0.002	0.007	0.9995	0.389	0.393	98.98
Ni	0.002	0.007	0.9997	2.115	2.142	98.74
Cu	0.025	0.083	0.9992	11.98	12.22	98.04
Zn	0.032	0.106	0.9993	83.0	82.3	100.85
As	0.001	0.003	0.9998	0.070	0.068	102.94
Se	0.012	0.040	0.9992	0.1112	0.1152	96.53
Sr	0.09	0.030	0.9996	57.22	55.54	103.03
Cd	0.001	0.003	0.9997	2.831	2.876	98.44
Sb	0.002	0.007	0.9996	1.96 *	2.00 *	98.00
Pb	0.002	0.007	0.9996	0.19	0.20	95.00
Hg	0.001	0.003	0.9998	0.0276	0.0297	92.93

* Not present in the certified matrix. Element added for method validation.

**Table 4:** Trace elements (**a**), potentially non-toxic elements (**b**), and potentially toxic elements (**c**) present in dried spices. The contents are expressed as the mean value (mg kg ^−1^) and standard deviation.

(a)							
	Cr	Fe	Mn	Cu	Se	Zn	
A	0.04 ± 0.001	268.850 ± 12.750	15.87 ± 0.558	11.089 ± 0.380	0.072 ± 0.001	44.060 ± 1.946	
B	0.059 ± 0.000	101.221 ± 7.653	11.925 ± 0.045	10.014 ± 0.059	0.244 ± 0.002	31.539 ± 0.066	
C	0.119 ± 0.006	136.998 ± 9.447	627.263 ± 28.417	2.599 ± 0.104	0.011 ± 0.001	5.798 ± 0.332	
D	0.193 ± 0.002	303.000 ±10.300	14.839 ± 0.520	8.366 ± 0.023	0.254 ± 0.008	20.982 ± 0.100	
E	0.314 ± 0.015	251.211 ± 6.452	13.964 ± 0.538	6.837 ± 0.379	0.028 ± 0.001	30.157 ± 1.764	
F	0.021 ± 0.000	314.231 ± 6.921	9.114 ± 0.026	8.631 ± 0.095	0.052 ± 0.008	30.013 ± 0.267	
G	0.096 ± 0.003	13.736 ± 1.326	95.802 ± 0.455	5.879 ± 0.054	0.008 ± 0.001	6.085 ± 0.082	
H	2.357 ± 0.029	10.586 ± 2.531	85.896 ± 0.936	2.376 ± 0.080	0.003 ± 0.001	15.652 ± 0.013	
I	0.088 ± 0.044	86.554 ± 4.586	16.747 ± 1.330	2.871 ± 0.052	0.287 ± 0.003	35.357 ± 0.187	
L	1.113 ± 0.010	289.667 ± 10.441	19.505 ± 0.024	6.874 ± 0.023	0.254 ± 0.003	48.049 ± 0.035	
M	0.129 ± 0.007	192.395 ± 13.521	16.289 ± 0.607	10.266 ± 0.085	0.505 ± 0.009	26.638 ± 309	
N	0.152 ± 0.001	124.569 ± 5.114	10.536 ± 0.168	11.804 ± 0.037	0.485 ± 0.006	36.698 ± 0.148	
O	0.109 ± 4.813	95.142 ± 4.236	103.145 ± 4.813	6.321 ± 0.284	0.009 ± 0.001	6.843 ± 0.263	
**(b)**							
	**Sr**	**Ni**	**Sn**	**V**	**Co**	**Sb**	**Be**
A	17.404 ± 0.716	3.053 ± 0.123	0.052 ± 0.001	0.028 ± 0.001	0.052 ± 0.001	0.001 ± 0.000	<LOD
B	62.373 ± 0.023	0.416 ± 0.004	0.510 ± 0.019	0.084 ± 0.001	0.271 ± 0.001	0.004 ± 0.002	<LOD
C	36.705 ± 1.657	0.753 ± 0.034	0.108 ± 0.038	0.040 ± 0.001	0.048 ± 0.002	0.008 ± 0.008	<LOD
D	95.917 ± 0.197	0.892 ± 0.010	0.617 ± 0.017	0.138 ± 0.001	0.111 ± 0.001	0.006 ± 0.001	<LOD
E	7.479 ± 0.302	1.124 ± 0.069	0.126 ± 0.073	0.303 ± 0.013	0.094 ± 0.005	0.010 ± 0.001	<LOD
F	4.299 ± 0.021	0.639 ± 0.010	<LOD	0.021 ± 0.001	0.227 ± 0.001	0.003 ± 0.000	<LOD
G	8.667 ± 0.047	1.194 ± 0.009	1.174 ± 0.011	0.066 ± 0.001	0.020 ± 0.001	0.003 ± 0.002	<LOD
H	50.586 ± 0.127	2.766 ± 0.012	1.054 ± 0.028	0.052 ± 0.001	0.048 ± 0.001	0.013 ± 0.000	<LOD
I	9.414 ± 0.929	0.732 ± 0.045	0.804 ± 0.007	0.024 ± 0.001	0.053 ± 0.001	0.005 ± 0.001	<LOD
L	89.403 ± 1.563	2.087 ± 0.021	0.813 ± 0.006	1.415 ± 0.013	0.288 ± 0.004	0.016 ± 0.000	<LOD
M	15.222 ± 0.219	1.134 ± 0.011	2.392 ± 0.039	0.049 ± 0.006	0.132 ± 0.001	0.022 ± 0.016	<LOD
N	55.034 ± 0.490	0.928 ± 0.013	0.735 ± 0.013	0.062 ± 0.001	0.145 ± 0.002	0.003 ± 0.003	<LOD
O	9.547 ± 0.287	1.293 ± 0.061	1.439 ± 0.129	0.073 ± 0.001	0.022 ± 0.001	0.028 ± 0.034	<LOD
**(c)**							
	**Cd**	**Pb**	**As**	**Hg**		**Al**	
A	0.026 ± 0.001	0.034 ± 0.001	0.024 ± 0.002	0.01 ± 0.001		19.963 ± 0.742	
B	0.010 ± 0.000	0.023 ± 0.000	0.015 ± 0.000	0.01 ± 0.000		32.355 ± 0.008	
C	0.014 ± 0.000	0.034 ± 0.001	0.005 ± 0.000	0.003 ± 0.001		20.532 ± 1.097	
D	0.012 ± 0.001	0.043 ± 0.000	0.078 ± 0.001	0.003 ± 0.001		87.507 ± 0.269	
E	0.017 ± 0.001	0.096 ± 0.005	0.086 ± 0.002	0.003 ± 0.001		145.216 ± 6.281	
F	0.011 ± 0.000	0.010 ± 0.001	0.003 ± 0.000	0.002 ± 0.001		7.112 ± 0.108	
G	0.003 ± 0.000	0.008 ± 0.000	0.002 ± 0.000	0.003 ± 0.001		43.064 ± 0.253	
H	0.071 ± 0.001	0.023 ± 0.000	0.005 ± 0.000	0.002 ± 0.001		27.168 ± 0.547	
I	0.079 ± 0.001	0.012 ± 0.001	0.009 ± 0.000	0.002 ± 0.001		8.863 ± 0.390	
L	0.065 ± 0.000	0.544 ± 0.003	0.339 ± 0.006	0.004 ± 0.001		930.198 ± 5.269	
M	0.034 ± 0.002	0.026 ± 0.002	0.009 ± 0.002	0.004 ± 0.002		19.914 ± 2.904	
N	0.021 ± 0.001	0.015 ± 0.001	0.026 ± 0.001	0.003 ± 0.001		32.508 ± 0.006	
O	0.005 ± 0.002	0.011 ± 0.002	0.005 ± 0.002	0.002 ± 0.001		46.889 ± 1.678	

A, black cumin seeds; B, Iranian Tokhme Sharbati; C, clove buds; D, Shahjeera; E, Abbaszadeh safron; F, organic fenugreek; G, whole black pepper; H, cinnamon; I, Abthul Ahmar (Asario); L, Ajwan seeds; M, whole coriander seeds; N, black sesame seeds; O, Sabja seeds.

## Data Availability

Data is contained within the article.
